# Supplementary material for: Clinical outcome of FIGO 2018 stage IB3/IIA2 cervical cancer treated by neoadjuvant chemotherapy followed by radical surgery due to lack of radiotherapy equipment: A retrospective comparison with concurrent chemoradiotherapy
Source: PLoS One. 2022 Mar 24;17(3):e0266001. doi: 10.1371/journal.pone.0266001 (PMC8947074; doi:10.1371/journal.pone.0266001)
Supplement: Attachment — Submitted filename: Response to Reviewers.docx [file pone.0266001.s002.docx]

Dear editor,

We have received the comments on our manuscript entitled " Clinical outcome of FIGO 2018 stage IB3/IIA2 cervical cancer treated by neoadjuvant chemotherapy followed by radical surgery due to lack of radiotherapy equipment: A retrospective comparison with concurrent chemoradiotherapy " by Jing Zeng, Peisong Sun, Quanhong Ping, Shan Jiang, Yuanjing Hu. According to the comments of the reviewers, we have revised our manuscript. The revised manuscript and the detailed responses to the comments of the one reviewer are attached.

Sincerely yours,

Yuanjing Hu

**Reviewer #1: For the critical analysis of the article, I suggest the evaluation of the following topics:**

**- Address the clinical-pathological differences between the types of cervical cancer (SCC x AC / ASC) in the discussion, as the groups present statistical differences in this regard, which may interfere with the results obtained.**

**Reply:** Thanks for pointing out this problem. We have modified our text as advised (see Discussion, Page 17-18, line315-327) with yellow color.

“Furthermore, 45 patients with AC/ASC had low 5-year OS. Previous studies have also suggested that the AC/ASC patients with the same FIGO stage have poorer prognostic outcomes than SCC [36, 37]. According to the present guidelines [7, 38], the therapeutic strategy recommended for SCC and AC/ASC is the same. Surgery and radiotherapy are recommended as the therapeutic schedule for early-stage cervical cancer. In our study, 38 patients were treated by CCRT, and NACT-RS only treated seven. However, a population-based analysis of about 2,773 patients with early-stage adenocarcinoma (IB-IIA) found that surgery remains the optimal local treatment modality for these patients [39]. Another retrospective study of about 3,102 patients with cervical adenocarcinoma using the Epidemiology and End Results (SEER) database also found that surgery is an independent favorable prognostic factor for early stages patients [40]. Therefore, it is significant to make new clinical treatment strategies for cervical AC/ASC.”

**-Evaluate include in the discussion the recent data from the following article: DOI: 10.1038 / s41598-021-86786-y**

**Reply:** Thanks for pointing out this problem. We have modified our text as advised (see Discussion, Page 18, line323-327) with yellow color.

“Another retrospective study of about 3,102 patients with cervical adenocarcinoma using the Epidemiology and End Results (SEER) database also found that surgery is an independent favorable prognostic factor for early stages patients [40]. Therefore, it is significant to make new clinical treatment strategies for cervical AC/ASC.”

**Reviewer #2: Well writen, considering its a common cancer in low and moddle income countries we need to consider the possibility of unavaliable radiotheraphy service so it is important to consider other therapeutic possibilities. Patients and methods well described, Results good comparasion between the groups, toxocity, PFS and OS. Good discussion.**

**Reply:** Thanks you. This research was a small-sample retrospective study with a particular inherent bias. A larger prospective randomized trial with a multicenter is required to confirm these results.
